# Recombinant polypeptide production in *E. coli*: towards a rational approach to improve the yields of functional proteins

**DOI:** 10.1186/1475-2859-12-101

**Published:** 2013-11-01

**Authors:** Ario de Marco

**Affiliations:** 1 Department of Biomedical Sciences and Engineering, University of Nova Gorica (UNG), Glavni Trg 9 - SI-5261, Vipava, Slovenia; 2 Therapeutic Antibody Platform, Institut Curie, 3-5 Impasse Reille, Paris 75014, France

**Keywords:** Carrier protein, Chaperones, Expression rate, Fusion proteins, Inclusion bodies, Isomerases, Promoter, Regulative sequences, Secretion mechanisms

## Abstract

The development of complementary technologies enabled the successful production of recombinant polypeptides in bacteria and opened to biology researchers new avenues as obtaining suitable amounts of proteins necessary for their experimental work became easy, fast, and inexpensive. Nevertheless, the recombinant approach remained somehow unpredictable, since many constructs resisted to apparent production or accumulated as aggregates.

Several factors and physical/chemical conditions that could improve the accumulation of native-like protein were identified. At the same time, it was acknowledged that the outcome of most of them was erratic and that almost any protein required its own specific optimized set of conditions to achieve its correct folding. The attempt to understand the critical points specific for recombinant protein production missed the goal of setting universally useful protocols, but contributed to the increase of the rate of success by proposing always new empiric combinations.

Nevertheless, the results published in the recent literature allow for a better comprehension of some key mechanisms controlling protein production in *E. coli* and could enable the elaboration of rational methodologies for improving the quantitative and qualitative features of the produced polypeptides. This result will be achieved when the identification of the limiting step that impairs the accomplishment of the native folding for any single construct will become straightforward. This minireview will discuss how factors such as the expression rate, the folding machinery, and the secretion efficiency may impact the final protein yields.

## Introduction

The intuition that recombinant expression could represent a metabolic burden for the host cell arose already in the infancy of this technology [1]. Consequently, it was proposed to abandon the initial approaches that pushed the expression rates by exploiting high-copy number plasmids and strong (as well as leaky) promoters in favor of tighter control and slower expression rate obtained with suitable plasmids and reduced growth temperatures [2,3]. For instance, in the *lac* promoter-regulated system both the addition of glucose and the overexpression of the lacI repressor and of the T7 lysozyme repress gene expression leakage effectively, impairing negative effects on plasmid segregation during cell replication [4]. Recombinant expression can be regulated also by controlling the plasmid number per cell. This factor depends on the origin of replication sequences: whereas the pUC-based plasmids can accumulate in hundreds of copies per single cell, the pBR322-based plasmids produce tens of copies and the pSC101 and p15A systems just few.

**Figure 1:**
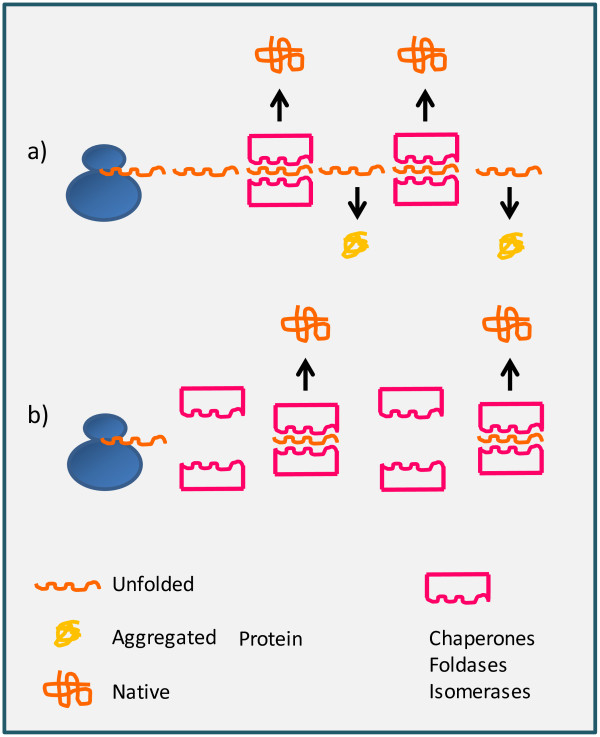
**Expression rate controls the protein native folding in the cytoplasm.** The recombinant polypeptide synthetized by the ribosomal machinery is mostly dependent on one/more chaperones and foldases to reach its native conformation. Disequilibrium between expression rate and folding capacity **(a)** will result in the accumulation of aggregates. This fate can be prevented by both reducing the expression rate of the recombinant protein and favoring the accumulation of chaperones and foldases **(b)**.

It was also observed that the induction of stress conditions by ethanol, benzyl alcohol or salt addition as well as by transient heat shock could improve the folding capacity of the treated bacteria [5]. All these perturbations stimulate the cell machinery specialized in protecting the macromolecule structure and the observed positive effect in terms of final recombinant protein yields was attributed to the accumulation of chaperones. This assumption was experimentally proven by demonstrating that chaperone overexpression could prevent the aggregation of recombinant proteins and it represented a significant biotechnological improvement [6]. Such approach has been successively optimized but still remains cumbersome due to the necessity of comparing several different chaperone combinations [7] (Figure 1). As an alternative, strains that could improve the production of a single target protein or a group of structurally similar proteins were generated by random mutagenesis [8-10]. Both deletion and overexpression of *E. coli* proteins resulted in the increased accumulation of recombinant proteins. The mechanisms involved vary from improved metabolism to the optimization of the protein quality control machinery [11]. The “Walker strains” [12] are spontaneous chromosomal mutants isolated by empirical selection of bacteria expressing toxic proteins and that were able to grow in the presence of both the expression inducer IPTG and ampicillin. They allowed for the recovery of several functional recombinant membrane proteins and created a wave of hope before the realization dawned that they were not miraculous, but constituted just another tool that could work in specific conditions. More interesting for the design of a more general methodology was probably the discovery of the molecular mechanism by which these strains managed to improve the recombinant protein yields.

### Expressing less to fold better

The Walker strains were introduced as “mutant hosts that allow the synthesis of SOME membrane and globular proteins” [12] and for more than ten years the reasons of this (partial) success remained unknown. It was surprising to discover that the mutations present in the different strains were indeed convergent and involved a single mechanism, namely they contributed to the reduced efficiency of the *lac* UV5 promoter that controls the expression of T7 RNA polymerase [13,14]. It was the first evidence that reducing the T7 RNA polymerase level in strains depending on this enzyme for recombinant expression could be a key element towards the control of the quality of the expressed protein and the increase of its accumulation. To assess such hypothesis, the control of the polymerase accumulation was obtained by means of the tunable expression of its inhibitor T7 lysozyme. The results confirmed that, in a specific range, there was an inverse correlation between polymerase availability and target protein accumulation [13]. This output is in agreement with the very early supposition that reducing the expression rate could limit the cell metabolic burden and promote protein folding quality [15]. The same effect of improved protein yield under conditions of promoter down-regulation was proved more recently for the arabinose-inducible expression system P(BAD) [16]. A spontaneous and less efficient mutant allowed for lower cell toxicity and higher productivity of secreted membrane proteins.

From a practical point of view, the synthesis of fewer polypeptides per cell would result in more correctly folded proteins because the cell machinery would work more effectively, i.e. within its limits. This condition avoids the accumulation of unfolded polypeptides that tend to aggregate and to produce a toxic effect. Clearly, not all of the proteins have the same requirements. For instance, it has been estimated that only 85 of all the *E. coli* proteins have strict GroEL-dependence for completing their folding [17], whereas 180 strongly depend on DnaK and a minority need both chaperones [18]. Therefore, the production of recombinant proteins that are either strictly GroEL-dependent or can use any/no chaperone to reach their native structure will exert a totally different pressure on the host cell in terms of competition for limiting components of the folding machinery. Both repressor leakiness and high expression rates can amplify the risk of accumulating misfolded proteins. The tight regulation of the recombinant expression is consequently crucial to limit the metabolic burden and the toxicity. From this perspective, it is interesting to learn that leakiness can be reduced 10-fold by a single mutation in the core domain of the *lac* repressor and that such mutation does not prevent IPTG binding and recombinant protein production [19]. A complementary approach should investigate what chaperones are necessary for any particular protein to fold correctly in its original eukaryotic cell with the aim of overexpressing the prokaryotic homologues in the recombinant system. For instance, a systematic work has identified the substrates of human Hsp90 [20] and such proteins could probably benefit from the co-expression of the *E. coli* orthologue chaperone ClpB when produced in bacteria*.*

### Minimal metabolic perturbation

The production of transcripts originating from a single gene copy of the target DNA integrated in the chromosome represents a solution for obtaining sufficient yields of heterologous protein and minimizing the metabolic burden for the host cells. In such conditions, the cell physiology is not perturbed by the presence of high-copy number instable plasmids and by the massive production of antibiotic resistance enzymes [21]. Sufficient protein yields are assured by enabling highly effective transcription and translation. Such result was obtained for the *XylS/Pm* expression system by combining several stimulatory elements in a unique expression cassette comprising the promoter, the 5′-untranslated mRNA region, and the XylS regulator coding sequence [22]. The synergic effect of single point mutations in the sequences which regulate the transcription and translation steps allowed improving the final protein yields of 75 times. Both increased transcript production and its improved translation contributed to the final protein accumulation [23]. Interestingly, the optimal level of the stimulation was protein-specific and higher transcriptional levels did not automatically correspond to higher yields of functional protein. These results were explained as the effect of the post-translational folding machinery saturation, a bottleneck that caused protein misfolding and aggregation. Such example underlines again how the final yields always depend on the limiting step in the long chain of events that leads from transcription to correctly folded proteins.

### Stoichiometric limitations

**Figure 2:**
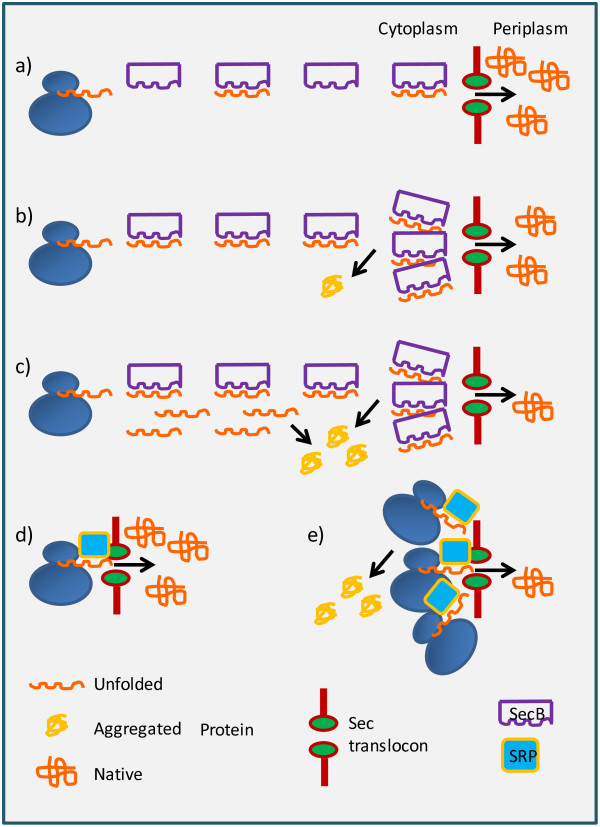
**Expression rate determines the folding rate of secreted proteins.** In optimal conditions **(a)**, the newly synthetized polypeptides can be successfully delivered to the translocon by the SecB co-chaperone and transported for folding into the periplasm. At higher expression rates, the polypeptides will progressively aggregate because either the Sec translocon **(b)** or/and SecB **(c)** will become limiting. The co-translational SRP-based secretion route could overcome the SecB paucity **(d)**, but shares the same export machinery and, therefore, any construct in excess to the translocon capacity will remain trapped in the cytoplasm **(e)**.

The question as to elevated expression rates can result in low yields finds its answer in the observation that proteins, in the majority of cases, depend on molecular factors to reach their functional structure. These factors can be as different as foldases, chaperones, interacting partners in the case of larger complexes, or membrane proteins involved in cell trafficking. The efficiency of the overall process leading to the production of a fully active protein relies on the availability of all the essential partners. Therefore, it is necessary to identify the critical molecular element involved in the folding process of a specific polypeptide that is the first to become limiting. This allows concentrating on its overproduction without causing the unaffordable energetic expense that the indiscriminate production of any foldase would cause to the cell. For instance, a specific chaperone overexpression is meaningful only when the recombinant protein requires their massive support to fold correctly, otherwise it represents a metabolic competition for the target recombinant protein [7]. In the case of secreted recombinant proteins, the Sec-translocon appears a major bottleneck because its carrier capacity can be easily saturated, as illustrated by Schlegel et al. [24]. As a consequence, unfolded protein intermediates accumulate in the cytoplasm and precipitate into aggregates (Figure 2). When toxic, these impair correct cell growth and division, leading to diminished biomass formation. The reduction of the recombinant translational activity can restore the physiological conditions and promote the periplasmic accumulation of processed and correctly folded heterologous proteins. This accomplishment recalls the results obtained with a library of vectors used to produce secreted proteins the components of which possess translational strengths covering a 10-fold range [25]. It was obtained by random alteration of the translation initiation regions of the signal sequence and the resulting vectors were tested in combination with a set of heterologous proteins. The results showed that the translation rate optimum is narrow and protein-specific. There is an upper limit above which the production drops due to the saturation of the system and a lower limit below which the protein synthesis machinery is not completely exploited.

In the past it was also demonstrated that secreting thermodynamically favored constructs by the co-translational SRP route could facilitate the production of functional proteins because this option avoided the SecB-dependent accumulation in the cytoplasm of partially folded, inactive or toxic intermediates [26,27]. Also the deletion of the gene responsible for the expression of the Trigger Factor (TF) chaperone contributed to limit the cell toxicity and to increase the accumulation of recombinant SRP-dependent proteins [16,28]. Since TF binds 1:1 the polypeptides emerging from the ribosomes, it was argued that TF competes with the signal recognition particle for the nascent proteins. Therefore, in the absence of TF the cytoplasmic accumulation of non-productive protein intermediates would be drastically reduced. Nevertheless, too elevated expression rates would finally result in secretion impairment independently of the deletion strategy and of the chosen export route, since SecB and SRP share the same translocon unit [24] (Figure 2).

Summarizing, the improvement of the recombinant disulfide bond-dependent protein production relies on the capacity of calibrating the secretion and the translocon rates. The illustrated data emphasize also the limits of production strategies depending on the periplasmic accumulation of heterologous proteins. Even after successful export through the Sec-translocon, the proteins will undergo molecular crowding in the reduced periplasmic space and will be prone to aggregation [29].

**Figure 3:**
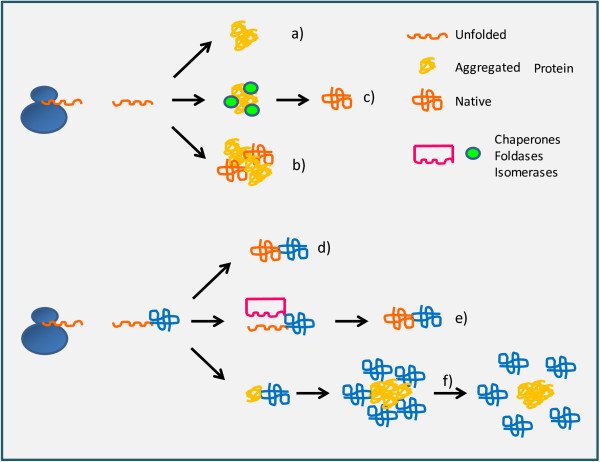
**Characteristics of the recombinant protein aggregates.** Recombinant polypeptides can aggregate in the inclusion bodies that are either completely inactive **(a)** or trap different amounts of active protein **(b)**. Furthermore, the polypeptides can be released from the inclusion bodies and a network of chaperones and foldases/isomerases can convert misfolded constructs into native proteins **(c)**. Highly soluble carriers fused to the target proteins can simplify the purification step **(d)** or keep in solution thermodynamically unfavorable partners for the time necessary to the folding machinery to complete its work **(e)**. Nevertheless, the partner can finally fail to fold, remaining (partially) misfolded. Such misfolded domains exposing hydrophobic residues tend to form micelles that are kept in solution by the soluble carriers that constitute an external hydrophilic layer **(f)**. Such soluble aggregates can be used to deliver the cargo in vivo and the carriers can be cleaved off when the biologically relevant target is reached.

The consequence is that the yields of periplasmicaly accumulated recombinant proteins remain low. A theoretical possibility exists of folding the (disulfide-independent) proteins in the bacterial cytoplasm, where the folding machinery is more effective, and then of exporting them into the periplasm by means of the Twin-arginine translocation pathway. The factors regulating this export route have been recently investigated [30] and it can have some biotechnological applications, such as the possibility of evaluating the correct folding of GFP-fused proteins [31]. Disulfide-bond dependent proteins can be forced to fold in the cytoplasm of mutated bacteria (oxidizing strains such as AD494 or Origami) in which the reducing pathways have been partially or totally impaired [32]. Others have proposed the secretion of such proteins into the medium [33,34]. Nevertheless, controlling the parameters that regulate the protein accumulation in the medium is cumbersome and the conditions can critically change when productions are scaled-up due to different leakage rates [34]. Concerning the “oxidizing strains”, the results are often deceiving in terms of yield [32,35,36], probably because the non-physiological conditions imposed to the cells slow down their growth. The overproduction of isomerases can help the correct folding of disulfide bond-dependent proteins. Nevertheless, DsbC isomerase used as a fusion tag is not sufficient to obtain reliable yields of functional target protein [32,36], even though it is statistically more effective than other carriers [36]. So far, the most innovative and complete approach for producing functional disulfide-rich proteins remains the model proposed by the Ruddock’ s group that enables oxidation and assisted folding by co-expressing both sulfhydryl oxidase and disulfide isomerase (DsbC or PDI) in the cytoplasm of wild type bacteria [37]. In the absence of sulfhydryl oxidase, most of the disulfide-dependent fusion proteins fused to DsbC that accumulated in the cytoplasm of wild type bacteria formed soluble aggregates [36] in which probably the (folded) carrier keeps in solution the aggregate target protein. According to the model initially proposed by Nominé et al. [38] (Figure 3), such soluble aggregates of fusion proteins are micelles with a core composed by tightly aggregated target proteins and an external hydrophilic layer formed of folded carriers. The amphiphilic nature of these structures has been recently harnessed for producing nanopills with bacterial binding capacity [39]. In the proposed model, the micelles are formed by conjugated proteins in which the external layer has the capacity to bind specifically the biological target. The cargo, composed by aggregated but partially active polypeptides, is then released by means of controlled enzymatic cleavage.

### Tuning protein aggregates

Aggregate formation is a constant of recombinant protein production. In contrast to previous hypotheses, we know now that aggregated proteins can conserve a consistent biological activity. Consequently, it is meaningful to study the aggregation process in vivo and to understand how chemical-physical factors can tune their characteristics [40] to improve their biotechnological value. Two opposite approaches have been proposed to cope with recombinant protein aggregation: try to limit it or to force the process and accumulate useful (functional and non-toxic) aggregates. The reversible dynamic of the aggregation-disaggregation process has been forced to improve the yields of soluble recombinant proteins by stimulating the bacterial cells to dissolve their inclusion bodies in vivo [7,41]. As an alternative, aggregation has been exploited as a valuable means for accumulating functional recombinant protein in the inclusion bodies and simplifying its purification for biotechnological applications [42] (Figure 3). From this perspective, the identification of genetic backgrounds favorable for the production of particularly dense inclusion bodies is justified by the interest in improving the protein processing, obtaining higher titers in fermentation and simplified purification steps due to the reduced volumes and faster sedimentation. Pandey et al. [43] induced the production of recombinant model proteins in a bacterial library of randomly obtained knock-out mutants and succeeded in isolating clones producing more/denser inclusion bodies by means of percoll density gradient centrifugation. Unluckily, the authors did not evaluate whether the protein packed into denser inclusion bodies had structural and functional characteristics comparable to the control material or have lost them, as it has been observed when lipase A was fused to the self-assembling peptide ELK16 to improve its precipitation [44].

The biological value of proteins trapped in the inclusion bodies can be related to their physical structure rather than to their functional features. For instance, controlling the fermentation parameters allowed for the obtaining of inclusion bodies with variable geometries. When used to treat surfaces, they facilitate the adhesion of eukaryotic cells and promote tissue culture and differentiation [45].

## Conclusions

Successful recombinant protein production is often strictly necessary to accumulate enough material for basic research studies as well as for biotechnological and clinical applications. A stimulating article [46] indicates that research stops where no reagents are available. Specifically, the authors remark that proteins that have a clear genetic link with human diseases, but for which reliable antibodies and chemical inhibitors are not accessible, are systematically less studied than similar proteins for which reagents are available. It would be interesting to analyze whether the reagent availability is related to the ease in obtaining functional and natively structured recombinant protein, since it can be expected that missing this step would represent a critical bottleneck. The knowledge accrued in the last 20 years conceives recombinant protein production as a long chain of successive events. Any of them can be limiting or can become the limiting after another obstacle has been removed. Therefore, the process optimization can be seen as a progressive removal of impairments: for a specific construct, too many transcripts blocks the translational machinery because there are not enough tRNA molecules for a specific codon, but in a strain overexpressing that tRNA the accumulated polypeptides can fail to fold due to DnaK shortage, and so on. Now we are probably approaching the comprehension of each single step involved in the whole process. For instance, several proteins that can improve protein secretion have been identified, the mechanisms by which low expression rate can lead to higher protein yields have been described in details, bacterial strains and vectors calibrated for working at physiological conditions are now available. The future efforts should be dedicated to a rational method to identify quickly and effectively the process weakness for any given protein to select the suitable counteraction. Combinatorial platforms exist for comparing experimentally protein production conditions by varying factors as different as bacterial strains, growth temperature, osmolyte concentration, fusion to different tags, promoter and transcription factor strength, mRNA stability and translation efficiency, chaperone co-expression, or signal peptide sequence [5,7,9,47-52]. By contrast, predictive models are still missing. The lack of searchable data might be one of the reasons and, consequently, it would be beneficial for the community to make the effort of collecting and annotating experimental data in a standardized manner to facilitate their metadata analysis. Certainly, this effort requires the input of extra energy and can be perceived as a limitation of the research freedom, both reasons for the scarce success of previous initiatives [53]. Nevertheless, such information is necessary to confirm some intuitions and to support statistically the cause-effect correlations evidenced by the already available results. The final accomplishment would be the development of holistic models able to consider all the elements that contribute to the protein production process.

## Competing interests

The author declares that he has no competing interests.
